# Correction to “Isolation of native EVs from primary biofluids—Free‐flow electrophoresis as a novel approach to purify ascites‐derived EVs”

**DOI:** 10.1002/jex2.81

**Published:** 2023-03-19

Preußer, C., Stelter, K., Tertel, T., Linder, M., Helmprobst, F., Szymanski, W., Graumann, J., Giebel, B., Reinartz, S., Müller, R., Weber, G., & von Strandmann, E. P. (2022). Isolation of native EVs from primary biofluids—Free‐flow electrophoresis as a novel approach to purify ascites‐derived EVs. *Journal of Extracellular Biology*, 1, e71. https://doi.org/10.1002/jex2.71

In the originally published article, there was an error in the assembly of panels in Figure 5d. The CD81 panel was duplicated and the CD63/CD9 panel was deleted. Figure 5 was revised accordingly showing the correctly assembled data panels and is shown below. This does not affect the scientific conclusions of the article.

Additionally, the following funding information should be included: Part of the work was funded by the HWMK, LOEWE research cluster iCANx.

We apologize for this error.

[Include the new figure here with caption]

Figure 5

**FIGURE 5:**
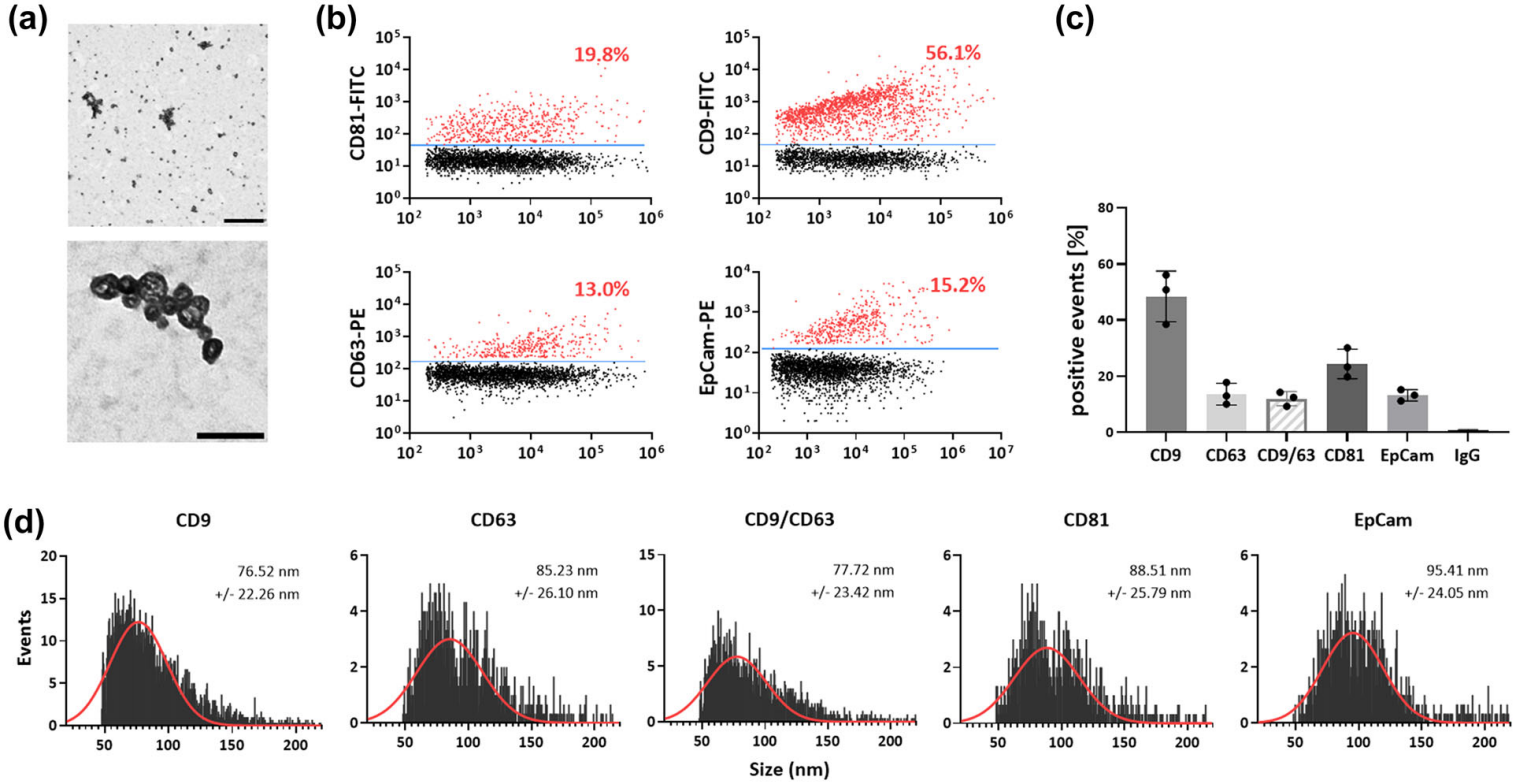


FFE allows purification of bona fide EVs from ascites. (a) Electron microscopy of purified vesicles. EVs were counterstained by uranyl acetate and are shown in two different magnifications. Scale bar, 500 nm (top); 100 nm (bottom). (**b)** Single‐particle phenotyping of ascites‐derived EVs. FITC‐coupled anti‐CD9 and anti‐CD81 and PE‐coupled anti‐CD63 and anti‐EpCAM antibodies at 12.5 nM were incubated with particles in a final volume of 50 μL freshly filtered PBS (0.1 μm ø). After staining, all samples were washed, ultracentrifuged, and suspended in the same volume (50 μL) of freshly filtered PBS (0.1 μm ø) before fluorescence measurement by nFCM. (c) Individual examples of phenotypic characterization of EVs from panel (c). Bivariate dot plots of the indicated fluorescence against SSC are shown. (d) Nanoflow cytometry analysis, depicting the size distribution (diameter) of particles positive for the indicated markers and indicating the mean of the size distribution calculated by Gaussian fit.

